# Toxic Epidermal Necrolysis and Graft-versus-Host Reaction: Revisiting a Puzzling Similarity

**DOI:** 10.1155/2013/651590

**Published:** 2013-06-03

**Authors:** G. E. Piérard, T. Hermanns-Lê, P. Paquet, A. F. Rousseau, P. Delvenne, C. Piérard-Franchimont

**Affiliations:** ^1^Laboratory of Skin Bioengineering and Imaging (LABIC), Department of Clinical Sciences, University of Liège, 4000 Liège, Belgium; ^2^University of Franche-Comté, 25030 Besançon, France; ^3^Department of Dermatopathology, Unilab Lg, Liège University Hospital, 4000 Liège, Belgium; ^4^Intensive Care Department and Burn Centre, Liège University Hospital, 4000 Liège, Belgium

## Abstract

Drug-induced toxic epidermal necrolysis (TEN) and acute cutaneous graft-versus-host reaction (GVHR) under immunopreventive therapy share some histopathological resemblance. So far, there are no serum biomarkers and no immunohistochemical criteria distinguishing with confidence and specificity the skin lesions of TEN and GVHR. Both diseases present as an inflammatory cell-poor necrotic reaction of the epidermis. This report compares three sets of 15 immunostaining patterns found in TEN, GVHR, and partial thickness thermal burns (PTTB), respectively. Three series of 17 skin biopsies were scrutinized. Irrespective of the distinct causal pathobiology of TEN and GVHR, similar secondary effector cells were recruited in lesional skin. Burns were less enriched in cells of the monocyte-macrophage disease. These cells likely exert deleterious effects in TEN and GVHR and cannot be simply regarded as passive bystanders. These life-threatening conditions are probably nursed, at least in part, by macrophages.

## 1. Introduction

Toxic epidermal necrolysis (TEN), formerly called under its eponym Lyell's syndrome, is a severe cutaneous adverse reaction (SCAR) to drugs [1]. Conceptually, both TEN and the Stevens-Johnson syndrome (SJS) are likely part of the same continuum of clinical presentations [2–6], and they are regarded to be likely distinct from erythema multiforme [7–9]. The precise TEN pathomechanism remains unclear [1, 5, 10–12]. Some toxic metabolites and/or cytotoxic inflammatory cells induce epithelial apoptosis and necrosis [5, 13–15]. Cytotoxic lymphocytes, regulatory T cells (Treg), macrophages, and dermal dendrocytes (DD) are likely involved, and they probably represent more than passive bystanders [16–20]. 

Graft-versus-host reaction (GVHR), both in its acute and chronic stages, is responsible for both potentially severe morbidity and mortality [21, 22]. GVHR remains quite frequent in susceptible groups of patients as about half of recipients of allogeneic hematopoietic cell transplantation (HCT) develop GVHR [23–25]. This condition results from a complex and intricate pathobiology sustained by interactions between the donor and host innate and adaptive immune responses. A number of lymphocyte subsets (naive, memory, Treg, Th1, Th17, NK, …) are involved, as well as eosinophils, mesenchymal stem cells and the monocyte-macrophage lineage including Factor XIIIa+ DD [26–30].

There is an overlap in a series of histopathological signs between early GVHR and various other posttransplantation diseases including some viral exanthems, immune reconstitution rash, and drug reactions [22]. The key points are keratinocyte apoptosis and satellite cell necrosis [31–35]. In absence of specific GVHR histopathological features, the value of skin microscopy remained fairly limited for landing support to the GVHR diagnosis or for ruling out other unrelated diseases. However, targeted immunohistochemistry was occasionally reported to bring some decisive clues [26, 36–42]. Both cell differentiation and tensegrity (shape) are better highlighted using some relevant immunopathological markers. It remains that some patients with GVHR develop a drug-induced TEN or a GVHR-related TEN-like lesion [43–46]. In some instances, the histopathological distinction between both conditions remains notoriously difficult or impossible to establish [27].

The aim of the present retrospective study was to revisit the input of immunopathology in the diagnosis of and distinction between cutaneous GVHR and TEN. The study was focused on the histopathology of incipient erythematous GVHR and TEN lesions with epidermal necrosis. Recent partial thickness thermal burns (PTTB) were used as control for epidermal destruction following physical injury.

## 2. Subjects and Methods

The study was approved by the Liège University Hospital and the local Ethics Committee of the Percy Military Teaching Hospital in Clamart. Three series of 17 lesional skin biopsies collected from untreated patients were retrieved from our files. They had been diagnosed as GVHR, TEN, and PTTB, respectively (Table 1). In each case, the epidermis was still present and in close contact with the dermis. Sections (5 *μ*m thick) were cut from formalin-fixed paraffin-embedded biopsies and stained by hematoxylin and eosin. Since clues from histopathology frequently fail to discriminate with confidence cutaneous GVHR from SCAR, immunohistochemistry targeting inflammatory cells, keratinocytes, and dermal cells was used aiming at better discriminating the three conditions. Paraffin sections were used for the avidin-biotin peroxidase method. After a 1-hour incubation time with each of the 15 primary antibodies (Table 2), slides were washed in Tris-buffered saline (TBS) and incubated for 30 min with the secondary antibody (biotinylated swine anti-rabbit, 1 : 300, Dakopatts). Slides were rinsed in TBS and covered by the EnVision (Dakopatts, Glostrup, Denmark) polymer-based revelation system. After TBS washing, Fast Red (Dakopatts) was used as chromogen substrate. The last steps consisted of counterstaining with Mayer's hemalum. Negative immunohistochemical controls were performed by omitting or substituting the primary and the secondary antibodies of the laboratory procedure.

Quantitative assessments were performed using computerized image analysis (MOP videoplan Kontron, Eching, Germany). Assessments were performed in a band 0.6 mm thick of the most superficial part of the skin. Cell counts were expressed as medians per mm^2^ of tissue (epidermis or dermis) section. Statistical comparisons were performed between TEN and GVHR using the unpaired nonparametric Mann-Whitney *U* test. Similar comparisons were performed in each condition to assess the relative densities of lymphocytes and macrophages. A *P* value lower than 0.05 was considered statistically significant.

## 3. Results

### 3.1. Lymphocytes

T lymphocytes were scanty in the three conditions. Skin-infiltrating lymphocytes in GVHR were memory CD45RO+ T lymphocytes, as well as cells of the CD4+ and CD8+ lineages. T lymphocytes grossly exhibited similar patterns of distribution in TEN and GVHR. A majority of them were present along the dermoepidermal junction, and few of them were present inside the epidermis. These latter cells were predominantly CD8+ lymphocytes, whereas the dermal inflammatory infiltrate was predominantly composed of CD4+ cells. By contrast, rare T lymphocytes were identified in recent PTTB. They were predominantly clustered in a perivascular distribution.

The lymphocyte density showed large interindividual differences in the three conditions (Table 3). No significant difference was yielded between TEN and GVHR.

### 3.2. Macrophages

Mac 387+, CD68+, myeloperoxidase+, and lysozyme+ macrophages were present in both TEN and GVHR lesions (Table 3). They were located along the dermoepidermal junction and haphazardly dispersed in both the dermis and epidermis. By contrast, these cells were rare in PTTB.

 In each condition, the Mac 387+ macrophage density was significantly (*P* < 0.01) superior to the CD45RO+ lymphocytes.

### 3.3. Dermal Resident Cells

Factor XIIIa+ DD appeared numerous and plump in the superficial dermis of most TEN and GVHR (Table 3). By contrast, PTTB did not apparently alter the Factor XIIIa+ DD population. Of note, the CD34+ DD remained tiny and rare in the three conditions.

Versican filled up most dermal cells in TEN and GVHR. They appeared plump compared to those in PTTB. In each condition, they were evenly distributed in the superficial dermis.

### 3.4. Keratinocytes

L1-protein+ (Mac 387+) keratinocytes were present in all cases of TEN, GVHR, and PTTB irrespective of the histopathologic grades. The cytoplasmic staining was focal or diffuse throughout the epidermis layers, even when the dermal inflammatory infiltrate was scant. Similarly, an uneven to strong Mac 387+ immunolabeling of keratinocytes was present in all biopsies of TEN-altered skin. The L1-antigen was, however, expressed mainly in suprabasal layers of TEN epidermis, with more discrete involvement of basal keratinocytes. The majority of samples from apparently uninvolved skin in TEN patients expressed the L1-antigen in a patchy pattern inside the epidermis, at sites where inflammatory cells were scanty or absent. In PTTB, all keratinocytes showed an intense L1-antigen labeling.

 Ulex europaeus agglutinin UEA type I labeled the upper portion or the whole epidermis in the three conditions.

The CD44 var 3 immunoreactivity surrounded each keratinocyte and was not altered by any of the assessed skin conditions.

Glutathione S transferase (GST) *π* was disclosed in small clusters of superficial keratinocytes in TEN.

The Ki-67 labeling was absent or very low (<2% basal cells) in the three conditions exhibiting large areas of keratinocyte necrosis.

The cytokeratin (CK) 15 was disclosed in rare (<1%) basal cells.

## 4. Discussion

TEN is one of the most dramatic drug-induced SCAR [1]. This life-threatening disease is characterized by the extensive destruction of the epidermis and epithelial mucosae. Some clues suggest that TEN results from a specific alteration of drug metabolism in keratinocytes [11, 15, 47]. More than 100 different drugs are currently involved in TEN, but only a minority of them accounts for the vast majority of cases [8]. Molecular and morphologic features of apoptosis were demonstrated in TEN-involved keratinocytes during the initial stage of the disease [13–15]. The following phase of TEN is characterized by full-thickness epidermal necrosis. Hence, it is assumed that the TEN pathomechanism likely combines early apoptosis and late necrosis [13, 14].

GVHR is recognized by clinicopathological alterations in recipients of HCT or bone marrow transplantation [21, 22]. Both immunological and nonspecific phenomena contribute to the clinical aspects. GVHR is one of the major complications of HCT and is responsible for posttherapeutic morbidity, decreased quality of life, and mortality [21, 22]. GVHR is critically induced and maintained by donor immunocompetent cells that are particularly directed against epithelia showing fast renewal including the liver, gastrointestinal tract, and epidermis. 

In some instances, a clinicopathological overlap exists between aspects of GVHR and TEN. This mixed condition typically represents a puzzling diagnostic dilemma [27, 44, 46]. Histopathology of both conditions shows a sparse inflammatory cell infiltrate with keratinocyte apoptosis. Satellite cell necrosis has been thought to be a typical feature of GVHR, although the same aspect has similarly been recorded in some TEN cases.

Until recently, macrophages were unfrequently reported in TEN and GVHR, and they were not considered to play a pivotal role in the disease. However, biopsies from the liver, gut, and skin of patients with lethal GVHR showed a striking preponderance of CD68+ macrophages in the inflammatory infiltrate. They were variably reported to be more or less numerous than T lymphocytes. The macrophage preponderance was especially found in the most severely damaged skin areas [27]. Indeed, the monocyte/macrophage L1-protein (Mac 387) expression was reported in over 80% of GVHR, but their possible primary or secondary involvement still remains debated. The preponderant macrophage infiltrate occasionally observed might result from the antilymphocyte effect achieved by preventive immunosuppressive treatments that generally do not target macrophages. However, this possibility does not rule out that primary macrophage-mediated GVHR exists. In such instance, GVHR resembles the inflammatory reaction to imiquimod application of the skin [48].

Macrophages could tentatively serve as a clue distinguishing TEN and GVHR. When the immunosuppressive regimen to HSCT fails to perform adequately, cutaneous GVHR shows a predominance of T lymphocytes over macrophages. Nowadays, the histopathological presentation of GVHR has changed, with refinements in the biologic and pharmacologic prevention of HSCT adverse effects. As immunosuppressive regimen preferentially targets T lymphocytes, the inflammatory reaction becomes restricted to Mac 387+ macrophages. The latter cells always outnumber the CD68+, myeloperoxidase+, and lysozyme+ macrophages. They are at least as numerous as the combination of CD45RO+, CD4+, and CD8+ T lymphocytes in TEN and even more abundant in recent cases. Even when macrophages outnumber T lymphocytes in TEN, a pure macrophage infiltrate is never seen. Hence, an inflammatory infiltrate composed almost exclusively of Mac 387+ macrophages could be a clue for a lymphocyte-abated GVHR.

Factor XIIIa+ DD encompass distinct functional subtypes [49]. Their numbers in the superficial dermis are variable and appear increased in the vast majority of TEN cases [18]. The large number of Factor XIIIa+ DD and their plump appearance in the perilesional and lesional TEN skin suggest their activation during the initial steps of the disease. The CD34+ DD do not show similar stimulation signs. The Factor XIIIa+ DD ultrastructural aspects are similar in TEN and GVHR, showing enlarged endoplasmic reticulum, and phagocytosis of collagen fibres and mast cell granules [39]. It is inferred that both the number and aspect of DD do not help discriminating the two diseases. They provide an additional link between them. Similarly, the versican+ dendritic cells appear stimulated in TEN and GVHR.

Keratinocytes are clearly the main target cells in both TEN and GVHR. Several immunohistochemical markers identifying keratinocytes and their adhesion molecules have been proposed to reveal changes in the epidermis of these two conditions [27]. The 365-kDa L1-protein exhibits some antimicrobial properties. The name calprotectin was coined for it. It is specifically identified using the Mac 387 monoclonal antibody during routine immunostaining. It consists of three calcium noncovalently bound polypeptide chains. It is expressed in neutrophils, monocytes, and some reactive macrophages, as well as in mucosal epithelium and reactive epidermis. Keratinocytes in the vicinity of inflammatory cells or covering tumors frequently express the L1-protein, a condition which has been interpreted as a nonspecific marker of cellular stress. The L1-protein keratinocyte expression during GVHR probably discloses sublethal cell injury. Such a finding appears to be very sensitive, although it lacks specificity. Hence, epidermal L1-protein expression is not specific and cannot be used for distinguishing GVHR from TEN.

In the present study, immunohistochemistry directed to UEA-1, CD44 var 3, and CK15 did not reveal marked differences between TEN and GVHR keratinocytes. The number of Ki67+ keratinocytes engaged in cell proliferation was similar in these two conditions. By contrast, it was largely overexpressed in some PTTB cases engaged in a repair phase. By contrast, the GST*π* immunolabeling was more frequently overexpressed in TEN than in GVHR, and it was often absent in PTTB.

## 5. Conclusion

TEN, GVHR, and PTTB share in common extensive necrosis of the full-thickness epidermis. The induction mechanisms are strikingly distinct, involving a drug cytotoxic reaction in TEN, a lymphocyte-mediated destruction in GVHR, and a physical threat in PTTB. The clinicopathological differential diagnosis between TEN and acute GVHR is blurred by overlaps in the skin conditions. This retrospective study suggests that the macrophage/lymphocyte balance represents a clue for distinguishing both conditions. 

## Figures and Tables

**Table 1:** Patient demography.

Parameter	GVHR	TEN	PTTB
M/F	10/7	6/11	9/8
Age, years (mean ± SD)	35 ± 8	39 ± 6	37 ± 10

**Table 2:** Panel of antibodies.

Antigen/antibody	Dilution	Source
CD4	1 : 25	Dako
CD8	1 : 25	Dako
CD34	1 : 50	Becton Dickinson
CD44 variant 3	1 : 10	Menarini
CD45RO	1 : 100	Dako
CD68	1 : 200	Dako
Cytokeratin 15	1 : 1	Neomarkers
Factor XIIIa	1 : 100	Neomarkers
Glutathione S transferase *π*	1 : 100	Castra Lab.
Ki-67	1 : 100	Dako
Lysozyme	1 : 300	Dako
Mac 387	1 : 400	Dako
Myeloperoxidase	1 : 1000	Dako
Ulex europaeus agglutinin-1	1 : 2000	Sigma
Versican	1 : 500	Seikagaku Corp.

**Table 3:** Medians (ranges) of cell densities/mm^2^ in the epidermis and superficial dermis of TEN, acute GVHR, and PTTB.

Cells/marker	TEN	GVHR	PTTB
Dermal T lymphocytes			
CD45RO	8 (2–19)	13 (9–29)	5 (1–9)
CD4	4 (0–10)	9 (5–16)	2 (0–6)
CD8	2 (0–5)	3 (0–6)	1 (0–3)
Dermal macrophages			
Mac 387	17 (9–31)	19 (13–38)	6 (1–8)
CD68	6 (3–10)	9 (5–17)	1 (0–4)
Myeloperoxidase	4 (3–9)	3 (2–8)	0 (0–3)
Lysozyme	5 (3–8)	10 (3–16)	1 (0–5)
Dermal dendrocytes			
Factor XIIIa	48 (24–65)	61 (45–83)	52 (33–68)
CD34	9 (6–15)	12 (8–17)	12 (5–16)
Versican	15 (7–32)	19 (14–42)	8 (3–17)
Keratinocytes			
Mac 387	389 (206–551)	296 (157–319)	29 (20–39)
UEA-1	97 (33–197)	78 (12–94)	33 (6–81)
CD44 var 3	408 (396–569)	333 (315–378)	15 (12–218)
GST*π*	32 (0–95)	15 (3–61)	0 (0–4)
Ki67	6 (0–19)	9 (7–42)	15 (0–63)
CK15	3 (0–8)	1 (0–4)	4 (0–7)
